# Efficacy of Systemic Vitamin C Supplementation in Reducing Corneal Opacity Resulting from Infectious Keratitis

**DOI:** 10.1097/MD.0000000000000125

**Published:** 2014-11-14

**Authors:** Yong-Wun Cho, Woong-Sun Yoo, Seong-Jae Kim, In-Young Chung, Seong-Wook Seo, Ji-Myong Yoo

**Affiliations:** Department of Ophthalmology (Y-WC, W-SY, S-JK, I-YC, S-WS, J-MY), College of Medicine; and Gyeongsang Institute of Health Science (S-JK, I-YC, S-WS, J-MY), Gyeongsang National University, Jinju, South Korea.

## Abstract

The objective of this study was to determine the effect of vitamin C supplementation on reducing the size of corneal opacity resulting from infectious keratitis.

The study included 82 patients (82 affected eyes), admitted for infectious keratitis from January 2009 to August 2013, who were followed for more than 3 months. Patients were divided into control, oral vitamin C (3 g/d), and intravenous vitamin C (20 g/d) groups during hospitalization. Corneal opacity sizes were measured using anterior segment photographs and Image J program (version 1.27; National Institutes of Health, Jinju, South Korea) at admission, discharge, and final follow-up. The corneal opacity size used for analysis was the measured opacity size divided by the size of the whole cornea.

The corneal opacity size decreased by 0.03 ± 0.10 in the oral vitamin C group, 0.07 ± 0.22 in the intravenous vitamin C group, and 0.02 ± 0.15 in the control group. Intravenous vitamin C reduced the corneal opacity size more than oral vitamin C (*P* = 0.043). Intravenous vitamin C produced greater reduction in corneal opacity size in younger patients (*P* = 0.015) and those with a hypopyon (*P* = 0.036).

Systemic vitamin C supplementation reduced the size of corneal opacity resulting from infectious keratitis. Intravenous vitamin C was more beneficial than oral supplementation, especially in younger patients and those with hypopyon.

## INTRODUCTION

Infectious keratitis remains a sight-threatening disease despite the development of potent new antibiotic agents and diagnostic techniques. Even with intensive antibiotic treatment, corneal damage can occur as a result of inflammatory processes caused by infection or scarring related to the healing process.^1,2^ The scarring that accompanies the resolution of infectious keratitis leaves many eyes visually impaired or blind.^3^ Thus, it is logical to employ strategies to reduce or prevent scar formation. Topical corticosteroids may seem like an obvious choice for this purpose, but their use is controversial. Some investigators advocate using topical corticosteroids along with antibiotics to reduce immune-mediated tissue damage and scarring. The Steroids for Corneal Ulcers Trial (SCUT) investigated the safety and efficacy of corticosteroids in the treatment of bacterial corneal ulcers.^4–6^ In SCUT, corneal scarring resulting from bacterial keratitis was noted to improve over time with corticosteroid treatment; the density of the scar-related opacity improved, leading to a concurrent improvement in vision.^7^ However, corticosteroids may also significantly slow the process of corneal wound healing, prolong infection, and predispose to stromal thinning and perforation.^6–8^ Therefore, treatments to reduce corneal scar formation without adverse effects, such as delayed re-epithelialization or perforation, are sought.

Corneal wound healing is a complex process, involving cellular changes and signaling molecules from cells of every layer of the cornea. Furthermore, a major component of the corneal haze in wound healing is because of changes in the composition and configuration of the extracellular matrix (ECM) and corneal neovascularization.^9,10^ Experimentally, vitamin C has been shown to play a role in synthesizing parallel arrays of ECM fibrils in cultured human keratocytes.^11^ Ascorbic acid is also known to be involved in the suppression of corneal neovascularization via its antioxidant effects and ability to enhance collagen synthesis.^12^ Therefore, we speculated that the administration of vitamin C may help to reduce corneal opacity caused by infectious keratitis.

The therapeutic effects of vitamin C were explored by Linus Pauling, who was the first to introduce the concept of high doses of vitamin C for the treatment of various conditions, from common cold to cancer.^13^ Since then, high doses of vitamin C have been widely used in the treatment and prevention of diabetes, atherosclerosis, common cold, cataracts, glaucoma, macular degeneration, stroke, heart disease, cancer, and other conditions.^14–17^

The purpose of the present study was to determine whether oral (3 g/d) or intravenous (20 g/d) vitamin C supplementation during hospital admission reduces corneal opacity resulting from infectious keratitis.

## MATERIALS AND METHODS

### Subjects

This study retrospectively reviewed the medical records of patients who were hospitalized from January 2008 to August 2013 for the treatment of infectious keratitis and followed for >3 months. All enrolled participants provided informed consent for this study. The review of patient charts providing data for this study was approved by the Institutional Review Board of the Gyeongsang National University Hospital (2012-09-003-001). Patients were excluded if they were pregnant or lactating; had a chronic systemic disease such as liver disease, renal disease, uncontrolled diabetes mellitus, or hypertension; had a history of renal calculi or gout; or underwent surgery during the current admission, including creation of a conjunctival flap, amniotic membrane transplantation, or keratoplasty (because of the high risk of impending perforation).

The participants were separated into 3 comparative groups, based on their vitamin C treatment. Group 1 received no vitamin C, group 2 received oral vitamin C (3 g/d), and group 3 received intravenous vitamin C (20 g/d).

Laboratory examinations included a complete blood count, erythrocyte sedimentation rate, C-reactive protein, electrolytes, blood urea nitrogen, creatinine, glucose, liver function tests, arterial blood gas analysis, urinalysis with microscopy, hepatitis B surface antigen, hepatitis B surface antibody, Venereal Disease Research Laboratory test, ABO typing, antibody screening test, prothrombin time, activated prothrombin time, human immunodeficiency virus antigen/antibody, hepatitis C surface antibody, pulmonary function tests, chest x-ray, and electrocardiography. These tests were repeated every 4 days to detect any complications that may occur after vitamin C supplementation.

### Main Outcome Measurements

**FIGURE 1:**
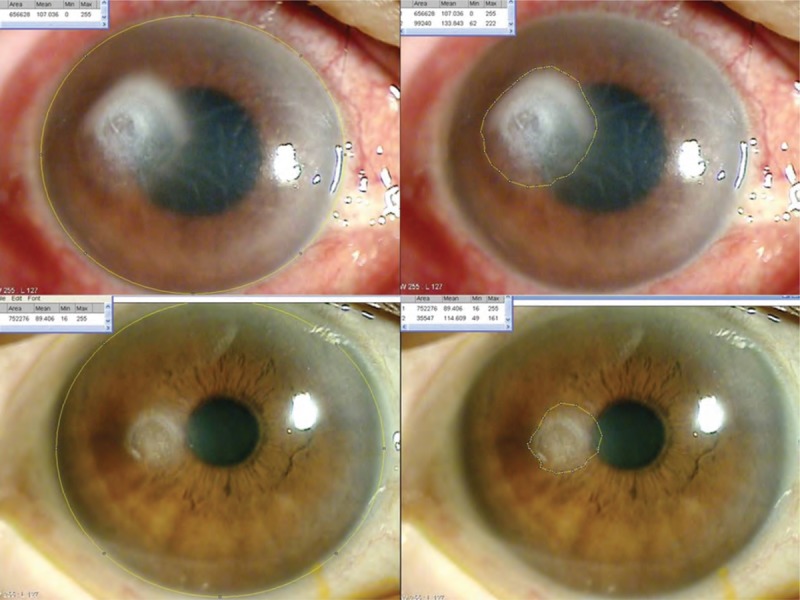
Measurement of corneal opacity size with Image J program (National Institutes of Health) (point-to-point check). Representative photographs of the anterior segment in a patient with a corneal ulcer at the initial assessment (top right, top left) and hospital discharge (bottom right, bottom left). Anterior segment photographs were obtained using a slit lamp with the eye in the primary position. The size of the whole cornea (top right, bottom right) and the corneal opacity (top left, bottom left) was determined using the Image J program with point-to-point check (yellow line).

Clinical data were collected at admission, discharge, and last follow-up visit. These data included age, sex, previous medical history including visual acuity, previous ocular history, intraocular pressure, location of corneal ulcer, and culture results from corneal specimens. Best corrected visual acuity was converted into logarithm of the minimal angle of resolution format (counting finger, 2.3; hand movements, 2.6; light perception, 3.0; no light perception 3.6). The size of the corneal lesion was measured using anterior segment photographs; all photographs were taken at the same magnification. The size of the corneal opacity was determined by point to point using the Image J program (version 1.27; National Institutes of Health). The opacity size was divided by the size of the whole cornea to facilitate comparisons (Figure 1).

### Vitamin C Supplementation

Participants were classified into control, oral vitamin C treatment, and intravenous vitamin C treatment groups. The latter 2 groups were administered either oral vitamin C (3 g/d) or intravenous vitamin C (20 g/d) throughout their hospital admission. For oral administration, patients were given 1 vitamin C 1000 mg tab (Korea Eundan Co, Ltd, Seoul, South Korea), containing ascorbic acid 1000 mg, 3 times/d. For intravenous administration, patients were given 1 ampoule of Merit C (Huons Co, Ltd, Seoul, South Korea), containing ascorbic acid 10 g/20 mL, 2 times/d. One ampoule of Merit C was added to 200 mL 5% dextrose in water for administration.

### Treatment of Infectious Keratitis

On admission, culture specimens were obtained by scraping the base and margins of the corneal ulcer. These specimens were placed on slides for Gram staining and potassium hydroxide (KOH) staining, according to standard protocols. Specimen samples were also inoculated onto blood, chocolate, MacConkey, and Sabouraud agar plate surfaces for culture. Bacterial and fungal isolates were identified using standard laboratory techniques.

All patients were treated with a standard antibiotic regimen immediately after admission and prior to the culture results. This consisted of hourly instillation of topical 0.5% moxifloxacin (Vigamox; Alcon, Fort Worth, TX). If a fungal infection was identified during KOH staining, an antifungal course was initiated, consisting of hourly application of topical 0.2% fluconazole, 0.15% amphotericin B, and 5% natamycin, with oral fluconazole. We changed the therapy as needed, based on the clinical response for each patient. When patients showed evidence of impending perforation, we performed conjunctival flap surgery, amniotic membrane transplantation, or keratoplasty and excluded the patients from the study.

### Statistical Analysis

All statistical analyses were performed using SPSS for Windows version 15.0 (SPSS Inc, Chicago, IL). Demographic characteristics and clinical factors that may influence corneal ulcer and corneal lesion size were compared using Levene Test for Equality of Variances. Changes in lesion size according to these factors were compared between the intravenous and oral vitamin C treatment groups using repeated measure analysis of variance. *P* values <0.05 were considered statistically significant.

## RESULTS

**FIGURE 2:**
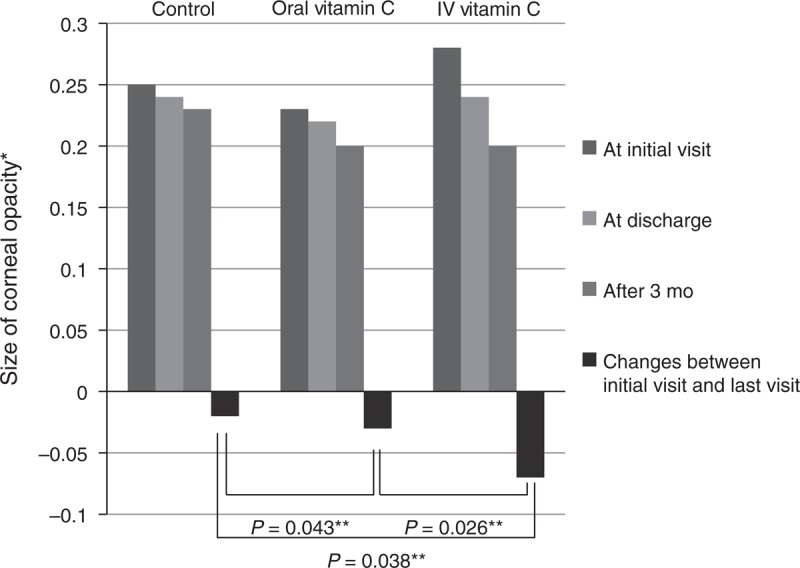
Comparison of the size of corneal opacity. This histogram shows that the size of the corneal opacity in all groups decreased 3 months after discharge compared with the size at the initial visit. The reductions were significantly greater in the oral and intravenous vitamin C treatment groups than in the control group. The reduction in corneal opacity size was significantly greater in the intravenous vitamin C treatment group than in the oral vitamin C group. IV = intravenous. *Measured opacity size divided by the size of the whole cornea. **Repeated measures analysis of variance.

**FIGURE 3:**
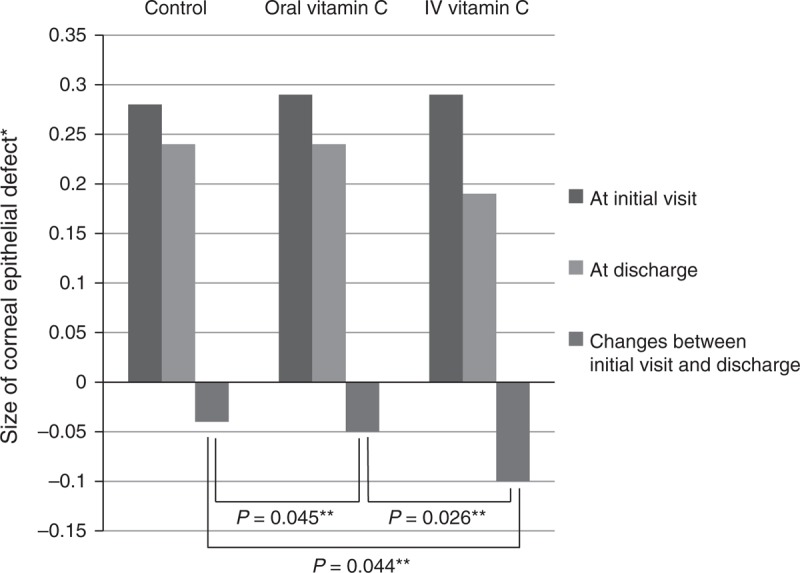
Comparison of size of corneal epithelial defect. This histogram reveals that the size of the epithelial defect decreased in all groups at hospital discharge compared with the size at the initial visit. The changes in epithelial defect size were significantly greater in the oral and intravenous vitamin C groups than in the control group. The reduction in the epithelial defect size was also significantly greater in the intravenous vitamin C group than in the oral vitamin C group. IV = intravenous. *Measured epithelial defect size divided by the size of the whole cornea. **Repeated measures analysis of variance.

Eighty two patients were enrolled in this study. Infectious keratitis was present unilaterally in all patients. The patients included 48 men and 34 women; their mean age was 64 ± 17 years. There were no statistically significant differences in baseline characteristics, such as the age, sex, visual acuity, intraocular pressure, previous ocular history, or duration of admission, between the 3 groups (Table 1). Of the 82 patients, 34 (44%) had positive results from culture study of the corneal specimens (Table 2). Positive corneal culture results were noted in 8 (40%) patients in the control group, 15 (41%) patients in the oral vitamin C group, and 11 (44%) patients in the intravenous vitamin C group. Of the risk factors that may affect corneal opacity size, the presence of hypopyon, location of ulcer, initial size of corneal infiltration, clinically suspected pathogen (bacteria, fungus, or other), and pathogen documented by positive corneal culture results were not statistically different between the 3 groups (Table 3). Furthermore, these risk factors were also not statistically different between the oral vitamin C and intravenous vitamin C groups (Table 4). The size of corneal opacity measured using anterior segment photographs at admission, discharge, and the end of the follow-up decreased by 0.03 ± 0.10 after oral vitamin C treatment, 0.07 ± 0.22 after intravenous vitamin C treatment, and 0.02 ± 0.15 in the control group (Figure 2). The decrease in corneal opacity size was significantly greater in the oral and intravenous vitamin C treatment groups than in the control group (*P* = 0.043 and 0.038, respectively; Figure 2). The decrease in corneal opacity size was also significantly greater in the intravenous vitamin C treatment group than in the oral vitamin C group (*P* = 0.026, Figure 2). From admission to discharge, the size of the corneal epithelial defect decreased by 0.04 in the control group, 0.05 in the oral vitamin C group, and 0.10 in the intravenous vitamin C group (Figure 3). The differences between the control and oral vitamin C groups and the control and intravenous vitamin C groups were statistically significant (*P* = 0.045 and 0.044, respectively) (Figure 3). The change in epithelial defect size was also significantly different between the oral and intravenous vitamin C groups (*P* = 0.026) (Figure 3).

**TABLE 1:**
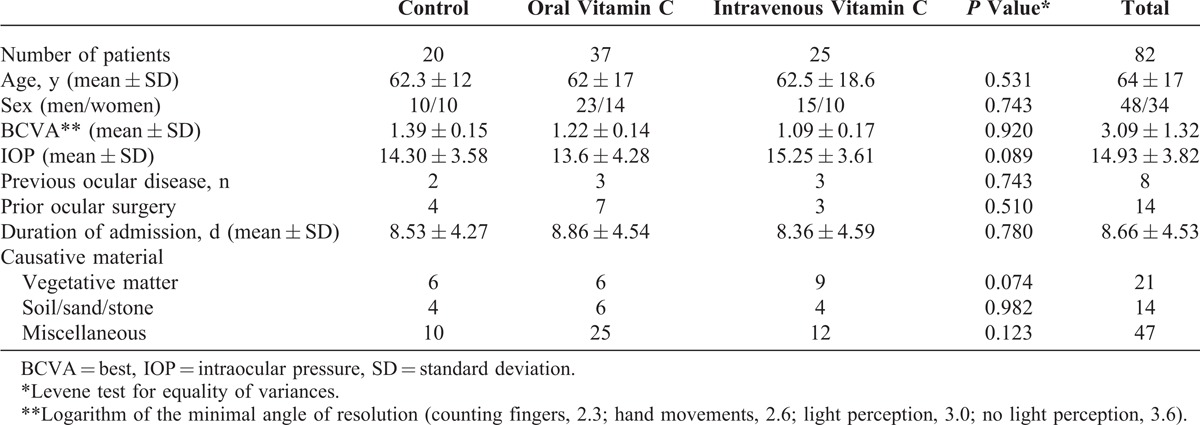
Baseline Subject Characteristics

**TABLE 2:**
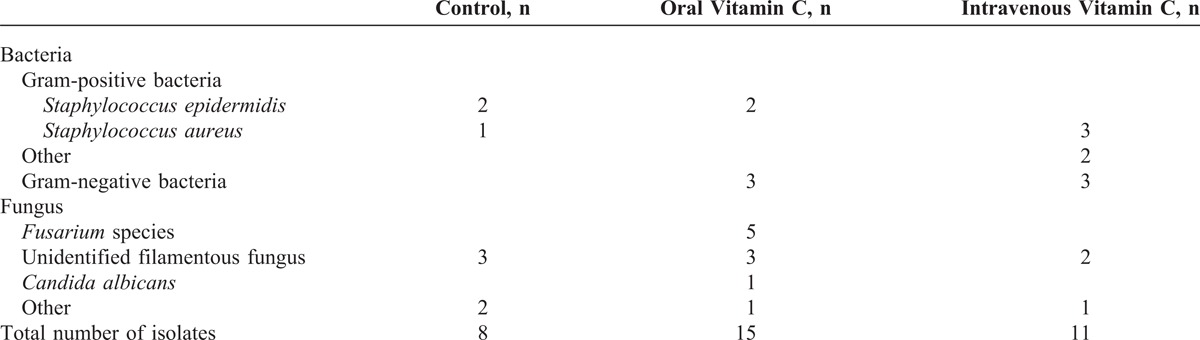
Organisms Isolated from Corneal Cultures

**TABLE 3:**
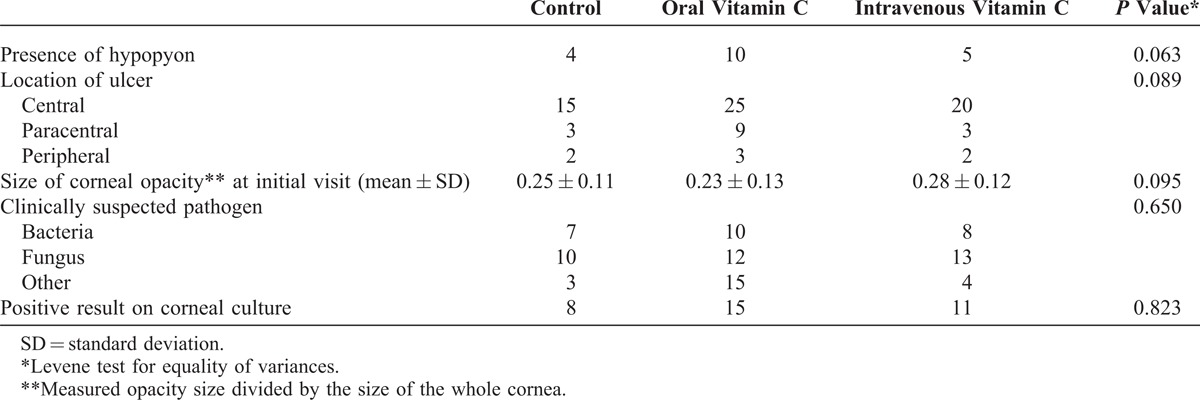
Factors Associated With Corneal Opacity Size in All Groups

**TABLE 4:**
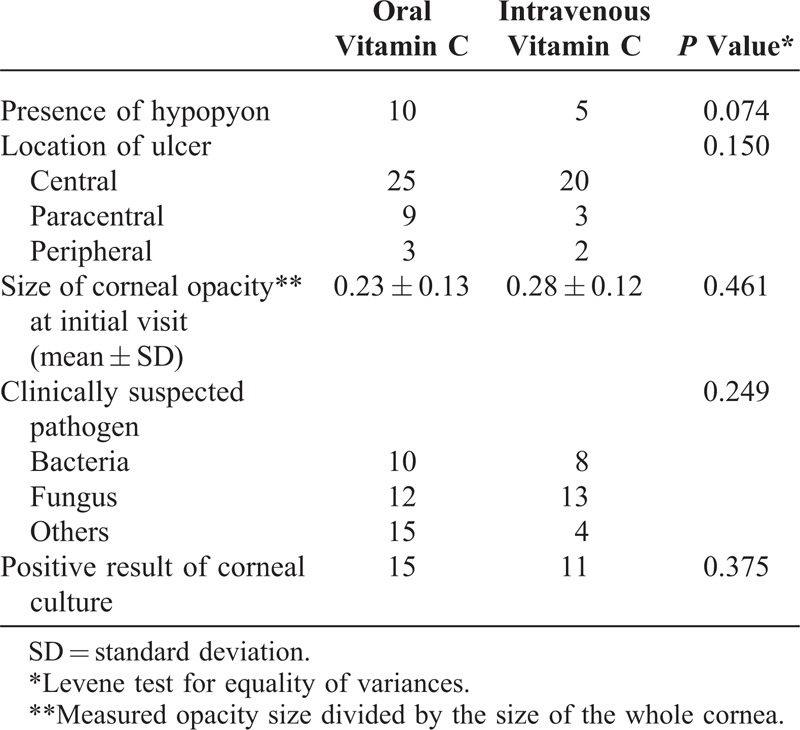
Factors Associated With Corneal Opacity Size in the Vitamin C Groups

Table 5 presents the results of univariate analysis of clinical risk factors that may affect the size of the corneal opacity for the 2 vitamin C groups. Comparing the intravenous vitamin C group with the oral vitamin C group demonstrated a significant reduction in corneal opacity size for infectious keratitis accompanied by hypopyon (*P* = 0.036) and patients <60 years (*P* = 0.015). Other clinical factors, such as age, previous ocular history, sex, duration of hospitalization, and pathogen (bacteria vs fungi), did not affect the decrease in corneal opacity size after vitamin C treatment.

**TABLE 5:**
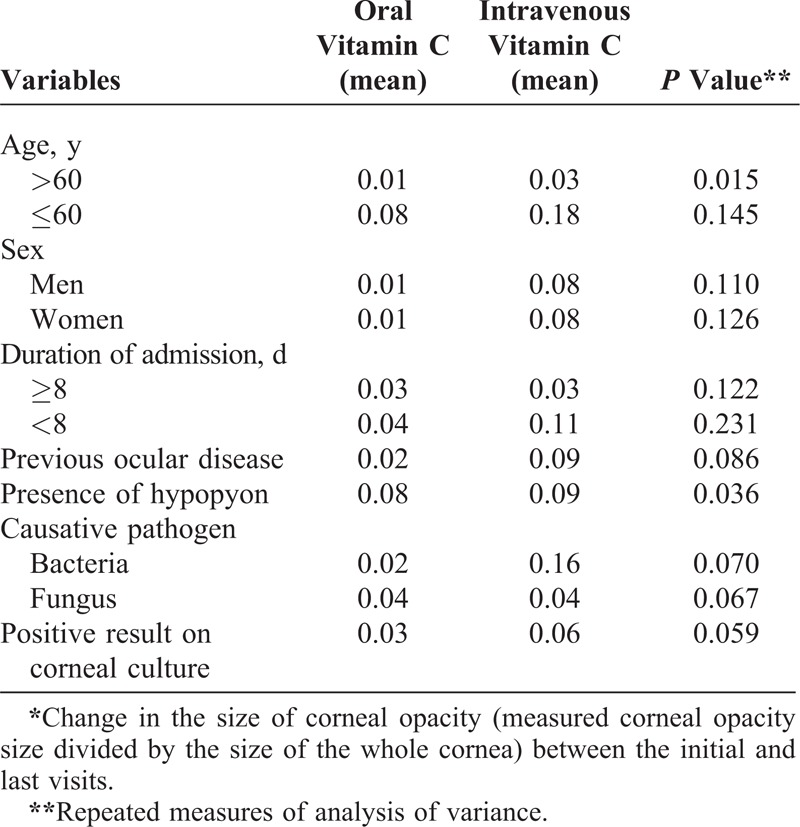
Factors Affecting the Change in Corneal Opacity Size* for the Vitamin C Groups

## DISCUSSION

The main finding of the current study is that systemic supplementation of vitamin C (oral or intravenous) effectively decreased the size of corneal opacities resulting from infectious keratitis. Furthermore, intravenous vitamin C was more effective than oral vitamin C in decreasing the corneal opacity size. Also, the reduction in corneal opacity size because of vitamin C treatment was greater when hypopyon was present and the patient was <60 years.

Corneal wound healing is a complex process of sequential synthesis and secretion of ECM components. For corneal injury because of infectious keratitis, the corneal epithelium initially heals by 3 sequential processes: migration, mitosis, and differentiation.^18^ If something goes awry in these healing processes, injured epithelial cells secrete specific growth factors or cytokines, such as tumor necrosis factor-α (TNF-α) and transforming growth factor-β1 (TGF- β1), depending on the degree of damage.^18–20^ These substances can affect adjacent epithelial or stromal cells. All layers of the cornea must heal to maintain original corneal transparency, but the layer that is most crucial to the development of corneal haze in response to injury is the corneal stroma.^9^ Once the corneal stroma is injured, keratocytes adjacent to the wound are activated and undergo myofibroblast transformation to repair the damaged stroma; contraction of the myofibroblasts results in an irregular contour of the healing scar, which may contribute to corneal haze.^21^ Moreover, several matrix metalloproteinases (MMPs) function in corneal wound healing and contribute to the initial tissue destruction after an injury.^19^ It is widely believed that the corneal opacity resulting from infectious keratitis is somewhat more serious than the postoperative corneal opacity after photorefractive keratectomy and Laser in situ keratomileusis surgery.^22,23^

Many investigations evaluating strategies to decrease corneal scar formation after treatment of infectious keratitis are currently in progress; SCUT is representative of these efforts.^4–7,24,25^ According to recent reports, the primary outcome of this trial revealed no benefit of adjunctive corticosteroids at 3 months after enrollment. Despite a possible delay in epithelial healing, no harm was observed with the use of corticosteroids.^6^ In the SCUT 12-month clinical trial, adjunctive topical corticosteroid therapy was shown to be associated with further improvement in long-term clinical outcomes in 5 patients with bacterial corneal ulcers not caused by *Nocardia* species.^7^ However, the use of topical corticosteroids in the treatment of bacterial keratitis continues to be controversial because of concerns about the potential for corticosteroids to exacerbate or prolong infection and/or delay corneal epithelial healing.^6^ Many investigators and clinicians have focused on treatments to decrease corneal opacity without these potential adverse effects. In the current study, our results suggest that vitamin C is a powerful candidate for reducing corneal opacity.

The direct mechanism whereby vitamin C reduces corneal opacity after infectious keratitis has not been established. To the best of our knowledge, this is the first report showing that systemic supplementation of vitamin C (oral or intravenous) effectively reduces the size of corneal opacity resulting from infectious keratitis. However, several reports have elucidated mechanisms by which vitamin C may affect corneal wound healing. First, vitamin C has been shown to accelerate the proliferation of corneal epithelial cells and the healing of epithelial defects.^26^ Impaired corneal epithelial cells around an infected wound release various inflammatory cytokines, such as TNF-α and TGF-β1, and these cytokines can worsen tissue necrosis.^19,20^ In our study, when the size of epithelial defects was compared between hospital admission and discharge, the differences between the control and oral vitamin C groups and the control and intravenous vitamin C groups were significant. Moreover, the change in epithelial defect size was significantly different between the oral vitamin C and intravenous vitamin C groups. One previous report described the protective effect of topically administered vitamin C on rabbit corneas after excimer laser keratectomy,^27^ and another report on rabbits noted that topical ascorbate significantly reduced the incidence of corneal ulceration and perforation after an alkali burn.^28^ Increasing the vitamin C concentration in the anterior chamber by systemic supplementation is expected to increase the vitamin C concentration in the corneal epithelium,^29^ thereby, promoting acceleration of corneal epithelial healing and reduction of corneal opacity. Another mechanism whereby vitamin C may reduce corneal opacity size is by its effects on collagen synthesis. Vitamin C is an important modulator of collagen production, acting as a cofactor for the hydroxylation of proline and lysine residues in procollagen. It enhances the production of several types of collagen in cultured rabbit keratocytes. Vitamin C is also known to suppress angiogenic factors, including vascular endothelial growth factor and MMPs, thereby inhibiting corneal neovascularization.^30–33^ Consequently, vitamin C may act as a cofactor of ECM synthesis and help damaged corneal stromal tissues recover in a normal manner. Finally, vitamin C exerts antioxidant and protective effects on the inflammatory response of the eye by scavenging reactive oxygen radicals and metabolites, such as myeloperoxidase, that are released by infiltrating inflammatory cells.^34–36^ Because vitamin C neutralizes oxygen-free radicals, it is expected to inhibit damage to neighboring epithelial and stromal tissues around a wound.^37^ Thus, vitamin C has a protective effect on both layers of the cornea and a preventive effect on the formation of corneal haze after infectious keratitis.

In our study, we compared the effectiveness of intravenously injected vitamin C with orally administrated vitamin C. Intravenous vitamin C decreased the corneal opacity more effectively than oral vitamin C. The reduction in opacity size was particularly greater when a hypopyon was present and the patient was <60 years. Since the presence of hypopyon represents severe inflammation, this suggests that vitamin C is particularly effective in more inflamed infectious keratitis, likely because of its anti-inflammatory and antioxidant effects.^12,37^ Elderly patients are known to have several systemic or ocular features that increase their vulnerability to inflammation, such as immunologic senescence, comorbidities (eg, lid abnormalities or conjunctivochalasis), and reduced corneal sensitivity. These features are risk factors of poor visual outcomes after infectious keratitis. Indeed, all 7 perforations (3 in the oral vitamin C group and 4 in the intravenous vitamin C group [data not shown]) occurred in patients >60 years of age, and the effect of vitamin C in older patients is limited because of age-related decreases in wound-healing activity. Therefore, the present study shows that vitamin C is more effective in reducing the size of corneal opacities in younger patients.

The findings of this study should be interpreted in the context of the following limitations. First, this study did not measure the concentration of vitamin C in the anterior chamber or serum. However, quantification of vitamin C may not be necessary because uptake of vitamin C by the active transporter of corneal epithelial cells has been reported to occur in a linear, concentration-dependent manner.^29^ Second, vitamin C treatment was only administered during the admission of the patients and did not continue after discharge from the hospital. Thus, we do not know whether the effectiveness of vitamin C treatment could have been further increased if it had been given for a prolonged period after discharge. Third, size measurements using the Image J program (National Institutes of Health) can be somewhat subjective, so interobserver variability may have occurred. In an attempt to compensate for this, we calculated the opacity size as a proportion of size of the whole cornea, and we conducted all measurements in a careful manner. Fourth, we measured the opacity size with slit lamp photographs; this method degrades the accuracy in measuring the size of the corneal opacity. So, future studies may use more accurate and precise ways to measure the size of corneal opacity, such as ConfoScan or cornea optical coherence tomography. Last, this study included a small number of subjects, and it was retrospective. Hence, further prospective studies with larger samples are required to clarify whether vitamin C reduces the size of corneal opacities resulting from infectious keratitis.

In conclusion, the results of this study showed that when used as an adjunct to antibiotic therapy, systemic (oral or intravenous) vitamin C supplementation has a beneficial effect on the healing process of infected corneas and reduces the size of corneal opacity resulting from infectious keratitis. Furthermore, intravenous vitamin C treatment decreased the corneal opacity size more effectively than oral vitamin C. In future, long-term clinical studies are warranted to establish the optimal dose, route of administration, duration of treatment, and frequency of administration of vitamin C for infectious keratitis.

## References

[1] Leibowitz HM Bacterial keratitis. In: Leibowitz HM ed. Corneal Disorders: Clinical Diagnosis and Management. Philadelphia: WB Saunders; 1984:353.

[2] Nordlund ML Pepose JS Corneal response to infection. In: Krachmer JH Mannis MJ Holland EJ eds. Cornea, Fundamentals, Diagnosis and Management. Philadelphia: Elsevier Mosby; 2005:95–114.

[3] Erie J Nevitt M Hodge D Incidence of ulcerative keratitis in a defined population from 1950 through 1988. Arch Ophthalmol. 1993;111:1665–1671. 8155038 10.1001/archopht.1993.01090120087027

[4] Srinivasan M Lalitha P Mahalakshmi R Corticosteroids for bacterial corneal ulcers. Br J Ophthalmol. 2009;93:198–202. 18829631 10.1136/bjo.2008.147298 PMC2632719

[5] Blair J Hodge W Al-Ghamdi S Comparison of antibiotic-only and antibiotic-steroid combination treatment in corneal ulcer patients: double-blinded randomized clinical trial. Can J Ophthalmol. 2011;46:40–45. 21283156 10.3129/i10-054

[6] Srinivasan M Mascarenhas J Rajaraman R Steroids for Corneal Ulcers Trial Group. Corticosteroids for bacterial keratitis: the Steroids for Corneal Ulcers Trial (SCUT). Arch Ophthalmol. 2012;130:143–150. 21987582 10.1001/archophthalmol.2011.315 PMC3830549

[7] McClintic SM Srinivasan M Mascarenhas J Improvement in corneal scarring following bacterial keratitis. Eye (Lond). 2013;27:443–446. 23238443 10.1038/eye.2012.270 PMC3597874

[8] Carnahan MC Goldstein DA Ocular complications of topical, peri-ocular, and systemic corticosteroids. Curr Opin Ophthalmol. 2000;11:478–483. 11141645 10.1097/00055735-200012000-00016

[9] Qazi Y Wong G Monson B Corneal transparency: genesis, maintenance and dysfunction. Brain Res Bull. 2010;81:198–210. 19481138 10.1016/j.brainresbull.2009.05.019 PMC3077112

[10] Chang JH Gabison EE Kato T Corneal neovascularization. Curr Opin Ophthalmol. 2001;12:242–249. 11507336 10.1097/00055735-200108000-00002

[11] Grobe GM Reichl S Characterization of vitamin C-induced cell sheets formed from primary and immortalized human corneal stromal cells for tissue engineering applications. Cells Tissues Organs. 2013;197:283–297. 23392073 10.1159/000346172

[12] Lee MY Chung SK Treatment of corneal neovascularization by topical application of ascorbic acid in the rabbit model. Cornea. 2012;31:1165–1169. 22832865 10.1097/ICO.0b013e318241433b

[13] Chambial S Dwivedi S Shukla KK Vitamin C in disease prevention and cure: an overview. Indian J Clin Biochem. 2013;28:314–328. 24426232 10.1007/s12291-013-0375-3 PMC3783921

[14] Vitetta L Sali A Paspaliaris B Megadose vitamin C in treatment of the common cold: a randomised controlled trial. Med J Aust. 2002;176:298–299. 11999270 10.5694/j.1326-5377.2002.tb04393.x

[15] Ichibe Y Ishikawa S Optic neuritis and vitamin C. Nihon Ganka Gakkai Zasshi. 1996;100:381–387. 8651057

[16] Iqbal Z Midgley JM Watson DG Effect of oral administration of vitamin C on human aqueous humor ascorbate concentration. Zhongguo Yao Li Xue Bao. 1999;20:879–883. 11270984

[17] Blanchard J Tozer TN Rowland M Pharmacokinetic perspectives on megadoses of ascorbic acid. Am J Clin Nutr. 1997;66:1165–1171. 9356534 10.1093/ajcn/66.5.1165

[18] Waring GO III Bouchard CS A matrix of pathologic response in the cornea. In: Krachmer JS Mannis MJ Holland EJ eds. Cornea. Philadelphia: Elsevier Mosby; 2011:47–82.

[19] Fini ME Girard MT Matsubara M Unique regulation of the matrix metalloproteinase, gelatinase B. Invest Ophthalmol Vis Sci. 1995;36:622–633. 7890493

[20] Carrington LM Albon J Anderson I Differential regulation of key stages in early corneal wound healing by TGF-beta isoforms and their inhibitors. Invest Ophthalmol Vis Sci. 2006;47:1886–1894. 16638995 10.1167/iovs.05-0635

[21] Jester JV Petroll WM Cavanagh HD Corneal stromal wound healing in refractive surgery: the role of myofibroblasts. Prog Retin Eye Res. 1999;18:311–356. 10192516 10.1016/s1350-9462(98)00021-4

[22] Yulish M Beiran I Miller B Ascorbate prophylaxis with mitomycin-C for corneal haze after laser-assisted sub-epithelial keratectomy. Isr Med Assoc J. 2012;14:382–385. 22891401

[23] Serrao S Lombardo M Corneal epithelial healing after photorefractive keratectomy: analytical study. J Cataract Refract Surg. 2005;31:930–937. 15975458 10.1016/j.jcrs.2004.12.041

[24] Sy A Srinivasan M Mascarenhas J Pseudomonas aeruginosa keratitis: outcomes and response to corticosteroid treatment. Invest Ophthalmol Vis Sci. 2012;53:267–272. 22159005 10.1167/iovs.11-7840 PMC3292362

[25] Lalitha P Srinivasan M Rajaraman R *Nocardia keratitis*: clinical course and effect of corticosteroids. Am J Ophthalmol. 2012;154:934–939. 22959881 10.1016/j.ajo.2012.06.001 PMC3498612

[26] Hayes S Cafaro TA Boguslawska PJ The effect of vitamin C deficiency and chronic ultraviolet-B exposure on corneal ultrastructure: a preliminary investigation. Mol Vis. 2011;17:3107–3115. 22171156 PMC3235536

[27] Kasetsuwan N Wu FM Hsieh F Effect of topical ascorbic acid on free radical tissue damage and inflammatory cell influx in the cornea after excimer laser corneal surgery. Arch Ophthalmol. 1999;117:649–652. 10326963 10.1001/archopht.117.5.649

[28] Pfister R Paterson C Hayes S Effect of topical 10% ascorbate solution on established corneal ulcers after severe alkali burns. Invest Ophthalmol Vis Sci. 1982;22:382–385. 7061209

[29] Talluri RS Katragadda S Pal D Mechanism of L-ascorbic acid uptake by rabbit corneal epithelial cells: evidence for the involvement of sodium-dependent vitamin C transporter 2. Curr Eye Res. 2006;31:481–489. 16769607 10.1080/02713680600693629

[30] Shakiba Y Mostafaie A Inhibition of corneal neovascularization with a nutrient mixture containing lysine, proline, ascorbic acid, and green tea extract. Arch Med Res. 2007;38:789–791. 17845900 10.1016/j.arcmed.2007.04.006

[31] BenEzra D Griffin BW Maftzir G Topical formulations of novel angiostatic steroids inhibit rabbit corneal neovascularization. Invest Ophthalmol Vis Sci. 1997;38:1954–1962. 9331259

[32] Utoguchi N Ikeda K Saeki K Ascorbic acid stimulates barrier function of cultured endothelial cell monolayer. J Cell Physiol. 1995;163:393–399. 7706381 10.1002/jcp.1041630219

[33] Knowles HJ Raval RR Harris AL Effect of ascorbate on the activity of hypoxia-inducible factor in cancer cells. Cancer Res. 2003;63:1764–1768. 12702559

[34] Kasetsuwan N Wu FM Hsieh F Effect of topical ascorbic acid on free radical tissue damage and inflammatory cell influx in the cornea after excimer laser corneal surgery. Arch Ophthalmol. 1999;117:649–652. 10326963 10.1001/archopht.117.5.649

[35] Schein OD Phototoxicity and the cornea. J Natl Med Assoc. 1992;84:579–583. 1629921 PMC2571692

[36] Cejkova J Lojda Z The damaging effect of UV rays below 320 nm on the rabbit anterior eye segment. II. Enzyme histochemical changes and plasmin activity after prolonged irradiation. Acta Histochem. 1995;97:183–188. 7544941 10.1016/S0065-1281(11)80097-8

[37] Williams RN Paterson CA A protective role for ascorbic acid during inflammatory episodes in the eye. Exp Eye Res. 1986;42:211–218. 3709692 10.1016/0014-4835(86)90055-2

